# Acknowledgement to reviewers

**DOI:** 10.1530/EC-15-A1

**Published:** 2026-01-19

The editorial board wishes to thank the following individuals who have helped to review manuscripts for *Endocrine Connections* during 2025. Their assistance is gratefully acknowledged.

**Table:** 

A Abaci
D H Abbott
S Ahmadi
S Akin
M Akkaya
G Akkus
Ö B Aksu
K Alexandraki
B Alexandre
S R Ali
I Ali Khan
A Alieva
H Alkaissi
M Almeida
M Al-Musali
F Álvarez-Nava
I Alves
U Ambroziak
R A Anderson
A Angelousi
A Antoniou-Tsigos
M Aon
M Arosio
M F Arshad
A Asghar
P C-M Au
L Audi Castroneves
M K Auer
A Awan
E Azizan
Z Azmaiparashvili

R Baba
T A S S Bachega
A Bachelot
R Balasubranamian
M Banaszak-Ziemska
M Barbot
Z Belaya
D Bell
M Bellusci
M Bengtsen
A E Berezin
M Biggs
A Bikas
A Binder
C Bizzarri
M Bjerre
C S Blesson
J Bollerslev
M Bonomi
D Braslavsky
P Bruzzi
K Busiah
G Butler
S Byrum

J Calissendorff
A E Calogero
M Cappa
J-C Carel
F Carlomagno
P V Carroll
A Cassio
J M Castellano Rodriguez
F Castinetti
F Ceccato
G Ceolotto
T Cerit
F Cetani
M Cetiner
V Chalmantzi
H-Y Chang
Y-C Chang
P Chanson
D Chartoumpekis
C Chatzakis
J Chen
L Chen
S C Chen
W Chen
X Chen
H L Cheng
C K Cheon
B Chevalier
M-D Chiara
M-N Chien
L Chioma
S Y Cho
J-H Choi
Y-M Choi
C-K Chou
K W Choy
M Christou
G Chrousos
H L Claahsen-van der Grinten
K Coelho e Oliveira
O Cogulu
L Colangelo
P F Collett-Solberg
S Corbetta
L Cruz
Y Cui
K H Czarnecka

J Q Dai-ju
J Daleprane
C Dalla
A F Daly
R D'Ambrosi
F Dassie
G De Filippo
S De Leo
N de Silva
N L de Silva
S M C De Sousa
M Debono
K Demir
S Deng
A Deodati
A Dessens
M Detomas
M Dhanasekaran
A Diekman
M Doknic
H Doneray
H H Dong
J Dora
A Duda-Sobczak

K Edy
G Effraimidis
Z A Efstathiadou
A H Eid
O El Kawkgi
M ElFiky
Y Elhassan
H C Emeksiz
N Esfandiari
R Espersen

H Fabbri-Scallet
K-C Fan
S Fang
C Farquharson
M H Fishbein
L Fonseca
A Foppiani
P A Foster
M Fragoso
G Fu
T Fukuda

F Gabalec
E Gan
M A Ganie
Y Gao
K Gariani
V Genc
N Georgopoulos
E Gevers
M L Gheorghiu
P Giacobini
C Giavoli
A Gilani
M P Gillum
H Gleeson
A Glover
Z M Gluvic
H Goto
S Gowru
A Goyal
B Grant
W B Grant
C H Gravholt
S B Gribsholt
S Groß
M A Grytaas
H Guan
D Guensch
N R Guidorizzi
O O Gul
L Gunnesson
K Guo
T Guran
A Gurlek

A Hadi
G Haeusler
T Hakata
K Hamamatsu
E Hammer
S E Hannema
M S Hansen
J Hardfeldt
E Hatipoglu
B Hauser
J Hawley
K Hawton
S Heebøll
C Heinze
P Hindmarsh
P Holmager
G Homuth
K Horiuchi
L Horodincu
S Howard
G Hu
J Hu
L-M Hung
I Hussain

G Iatrakis
N N Imga
H Inaba
V Iotova
G Iskrov
L Iughetti
Y Iwasaki

B Jarzab
B Javorsky
M J Jeon
C Jimeno
Y Jing
G Johannsson
H Jones
R Joshi
C–C Juan

S Kabisch
S Kamalanathan
A B M Kamrul-Hasan
G Kanakis
D Karasik
N Karavitaki
J Kaur
T Kearney
M Keskin
M T Khan
S Khatiwada
D Kim
J Kim
J H Kim
J Klein
D T Klink
T Kocjan
P Komarnicki
A Kong
P Konstantakou
L Koorneef
S Kotschi
A Kotwal
M Kralik
I Kraljevic
N Krone
R E Krone
L Ku
N Kumar
F-C Kuo
A Kuś
H Kwon
A Kyriakou

A La Silvia
E Lamback
L Lande Werke
J Lebl
L Lechner
S Leibnitz
M Levy
W Li
Y Li
R Libianto
J Liimatta
C H Lin
K E Lines
M Lipsmeyer
E Liu
F-S Liu
Y Liu
Y-P Liu
S Livadas
T Londero
A A Lopez-Gonzalez
J D Lowman
C-H Lu
A Lucas-Herald
A Luger
D Lui
F O Lurquin

M Madeira
R K Mahat
B M Mahdi
P Majety
P Malandrino
Y H Mamoojee
A Maniatis
M Manrai
M Manzano
L Marina
R Marrington
N M Martin
M C Matei
A J Mather
N Matikainen
M Matsushiro
A McGowan
J McNeilly
E Memi
C Menale
L P Menon
M Mercado
W Merk
D Merke
E Mills
S Minabe
S Minisola
M C Miranda
S Mir-Bashiri
S P Mollan
J Moludi
S Monticone
R Moon
M Mordenti
K Moreno-Tamayo
S Mu
K Muir
H L Müller
J Munarin
E Murillo-Zamora
P Murray
R D Murray
T Mushtaq

A Naciu
K Nakuluri
E Nascimento
M S G Naz
C Newman
M Niedziela
M Nolde
A Nordenstroem
L Nordestgaard
T Novoselova
E N Y Nyarko

A Odle
K Okamoto
M Olejarz
C Ooi
M Opalinska
J Østergaard
S O'Toole
M Ouni
A S Ozgu-Erdinc

R N R Padidela
A Palasz
G Paltoglou
X Pan
D Papadimitriou
D T Papadimitriou
L Papanastasiou
R D Paparodis
T Pappa
M Parasiliti-Caprino
F Paris
J Parker
S A Paschou
B Passaia
S Patjamontri
D Pedersen
I Pelsma
S Pereira
C Philippoteaux
R Pishdad
J Pott
A A Putilov

J E Quiroz-Aldave

O Ragnarsson
A Raimann
H K Råket
T Randell
S Rao
G Ratti
V Raverot
M Read
J Rege
N Reisch
R A Rey
S Richter
A Richter-Unruh
N Rittig
K Rizzoti
R Robeva
V Rochira
L Ruck
J Rymuza

N Sachithanandan
E Saglam
M Şahin
K Sakaki
M Salerno
D E Sandberg
V Sarathi
N Sawicka-Gutaj
C Scaroni
K Schilbach
L Schock
R Scott
A Secco
M Sekiya
F Sesti
S Shah
I Shaheen
G M Shaikh
Z Shan
A Sharma
B Sharma
H Shen
S Shoelson
L Sieminska
J F Silva
F A Simmen
S Sivaraman
A Skalniak
A Smit
G Sokratis
G Sommer
M Sone
S Soneda
Z Song
D Springer
A Spyroglou
V Srinivasan
R Srivastava
M Stancampiano
C Stefanaki
A Stigliano
A Strandqvist
M E Street
J-b Su
N Sukor
J Sun
W Sun

K C B Tan
S B Tan Öksüz
L Tarancon-Diez
E Tas
M Terzolo
A Tessnow
M Theodoropoulou
I Tizianel
A A Toogood
G Tornese
P Touraine
A K Trivedi
S Turan
G Twig
P Tzoulis

B Ucan
G Ueland
G Å Ueland

N Valdés
E van Boxel
M van Faassen
P van Houten
E van Velsen
E Varaldo
D A Vassiliadi
M C Velarde
A Venkataraman
G Vila
G Voltan
D Vrachnis

A Walczyk
W Walker
C-Y Wang
L Wang
Q Wang
Y Wang
S M Webb
F Weber
B Wen
J Wen
S Wiegand
C L Wood
J Worthington
V Wu
Y Wu
M Xing
C Xu
F Xu
R Xu
S Xu

S Yakar
I Yamauchi
C Yang
J Yang
M Yang
Y Yang
Z Yang
A Yarahmadi
M Yavropoulou
M Ybarra
C B Yeh
J S Yoon
H Yoshida
I Yoshii
T Yoshiike
C-L Yu
K Yu
L Yu

P Zagana
M Zaidi
D H Zangen
E Zavala
H-q Zhang
J Zhang
J-l Zhang
L Zhang
M Zhang
Q Zhang
Y Zhang
H Zhao
P Zheng
G N Zografos
L Zolkiewski

